# Determination of Key Intermediates in Cholesterol and Bile Acid Biosynthesis by Stable Isotope Dilution Mass Spectrometry

**DOI:** 10.4137/aci.s611

**Published:** 2008-03-25

**Authors:** Tadashi Yoshida, Akira Honda, Hiroshi Miyazaki, Yasushi Matsuzaki

**Affiliations:** 1 Raffles Japanese Clinic, Singapore, Singapore; 2 Tokyo Medical University, Kasumigaura Hospital, Ibaraki, Japan; 3 Pharmax Institute, Kanagawa, Japan

**Keywords:** isotope dilution mass spectrometry, biomarker, cholesterol synthesis, bile acid synthesis, mevalonate, oxysterol

## Abstract

For more than a decade, we have developed stable isotope dilution mass spectrometry methods to quantify key intermediates in cholesterol and bile acid biosynthesis, mevalonate and oxysterols, respectively. The methods are more sensitive and reproducible than conventional radioisotope (RI), gas-chromatography (GC) or high-performance liquid chromatography (HPLC) methods, so that they are applicable not only to samples from experimental animals but also to small amounts of human specimens. In this paper, we review the development of stable isotope dilution mass spectrometry for quantifying mevalonate and oxysterols in biological materials, and demonstrate the usefulness of this technique.

## Pathways for Cholesterol and Bile Acid Biosynthesis

Cholesterol homeostasis in human is maintained by two input pathways, comprised of dietary absorption and *de novo* synthesis, and two output pathways, comprised of direct secretion from liver to bile and conversion into bile acids (Everson, 1992). The rate-limiting step in the *de novo* cholesterol synthesis is the conversion of 3-hydroxy-3-methylglutaryl-CoA (HMG-CoA) into mevalonic acid (MVA) by HMG-CoA reductase (HMGCR) (Dietschy and Brown, 1974). In contrast, the bile acid biosynthetic pathway is initiated by either hepatic 7α-hydroxylation or hepatic and extrahepatic 27-hydroxylation of cholesterol. The former is catalyzed by microsomal cholesterol 7α-hydroxylase (CYP7A1), the first and rate-limiting enzyme in the classic pathway, while the latter is catalyzed by mitochondrial sterol 27-hydroxylase (CYP27A1), a key enzyme in the alternative pathway (Vlahcevic et al. 1992). Bile acid synthesis by the classic pathway accounts for more than 90% of total bile acids in humans (Duane and Javitt, 1999) while less than 50% of total bile acids is produced by this pathway in rats (Vlahcevic et al. 1997) and mice (Schwarz et al. 1996). Therefore, the measurement of CYP7A1 activity is more important than that of CYP27A1 activity for the evaluation of bile acid biosynthesis in humans.

## Direct and Indirect Assays of HMGCR and CYP7A1 Activities

Since HMGCR and CYP7A1 are crucial enzymes in understanding whole body cholesterol metabolism, a great deal of effort has been made to develop suitable assay methods for these enzyme activities. The primary methods have the great disadvantage that an invasive tissue biopsy is necessary for direct determination of these enzyme activities in humans. To overcome this problem, plasma biomarkers for evaluation of these enzyme activities has been explored.

Plasma levels of MVA, the immediate product of HMGCR, were positively correlated with HMGCR activities in rat liver (Popjak et al. 1979). In humans, the plasma MVA concentrations reflected (i) increased rates of whole-body cholesterol synthesis by treatment with cholestyramine resin, and (ii) decreased rates of whole-body sterol synthesis after consumption of a cholesterol-rich diet (Parker et al. 1982 and 1984). In addition, plasma concentration of lathosterol, an intermediate in the late cholesterol biosynthetic pathway, was reported to reflect hepatic HMGCR activity (Björkhem et al. 1987a) as well as whole body cholesterol synthesis (Kempen et al. 1988) in humans.

As for bile acid biosynthesis, Björkhem et al. (Björkhem et al. 1987b) demonstrated that serum levels of 7α-hydroxycholesterol (7A) correlated well with the activities of CYP7A1 in patients with gallstones treated with cholestyramine. In addition, Axelson et al. (Axelson et al. 1988) measured serum concentrations of 7α-hydroxy-4-cholesten-3-one (C4), the product of the next reaction following 7α-hydroxylation of cholesterol, and showed that it was a good marker for CYP7A1 activity in humans (Axelson et al. 1991). It was subsequently reported that serum concentrations of 7A (Hahn et al. 1995) and C4 (Sauter et al. 1996) reflected not only CYP7A1 activities but also bile acid synthesis in humans.

## The Methods for the Quantification of MVA

Table 1 summarizes the previously described methods for the quantification of MVA in the liver (enzyme assay), plasma or urine. The primary methods for assaying HMGCR activity have utilized a RI technique that measures the radioactivity in [^14^C]MVA produced from [^14^C]HMG-CoA (Shapiro et al. 1969; Goldfarb and Pitot, 1971; Shefer et al. 1972). The methods have been used for the direct determination of enzyme activity but they are not applicable to the quantification of plasma or urinary MVA. In contrast, the following methods, i.e. radioenzymatic assay, enzyme immunoassay, gas chromatography-mass spectrometry (GC-MS), high-performance liquid chromatography (HPLC), liquid chromatography-mass spectrometry (LC-MS) and liquid chromatography-tandem mass spectrometry (LC-MS/MS), can measure not only enzyme activity but also MVA concentrations in plasma and urine.

### Radioenzymatic assay

Radioenzymatic assay of the plasma MVA concentration was reported by Popjak et al. (Popjak et al. 1979). The method depends on the phosphorylation of MVA with [γ-^32^P]ATP and MVA kinase to 5-[^32^P]phospho-MVA, and the subsequent isolation of the 5-[^32^P]phospho-MVA together with known amounts of added 5-phospho[^14^C]MVA by ion-exchange chromatography. The detection limit of their radioenzymatic assay was 1–2 pmol (148–296 pg) indicating that it was not adequate for determining MVA in small amounts of plasma. In addition, there was a safety concern due to the handling of radioactive materials.

### Enzyme immunoassay

In 1998, Hiramatsu et al. developed an enzyme immunoassay for urinary MVA using a specific monoclonal antibody against MVA (Hiramatsu et al. 1998). This method is not only simpler than the previously described radioenzymatic assay but also completely avoids the risk of radiation hazards. However, the limit of detection was not better than that of the radioenzymatic assay.

### GC-MS

In 1972, Hagenfeldt and Hellström attempted to determine MVA concentration in rat blood by using GC-MS (Hagenfeldt and Hellström, 1972). In this procedure, the MVA was extracted from the acid aqueous phase as the lactone. The lactonization increased the hydrophobicity of MVA, so that they could extract it into organic phase. The resulting extract was treated with diazomethane to convert the coexisting fatty acids into their methyl esters. The unchanged mevalonolactone (MVL) with diazomethane in the extract was quantified by GC-MS in electron ionization mode (GC-EI-MS). The peak corresponding to the retention time of MVL appeared large due to interfering materials, such as fatty acids. However, the MVL could be quantified selectively because MVL exhibited an intensive peak at m/z 71 in the spectrum, whereas all fatty acid methyl esters gave rise to the inherent peak at m/z 74 produced by the McLafferty rearrangement ion of the methyl ester. Since then, urinary MVA has been successfully quantified by a similar GC-EI-MS method described above (Woollen et al. 2001).

In the 1970s, the RI technique was the standard method for assaying HMGCR activity, but the handling of radiolabeled materials was a great disadvantage of this method. In 1978, Miyazaki et al. developed a new non-RI method for assaying HMGCR activity in rat liver microsomes or liver slices using [^2^H_3_]HMG-CoA as a substrate and GC-MS in chemical ionization mode (GC-CI-MS) (Miyazaki et al. 1978). In this method, the resulting [^2^H_3_]MVL was derivatized to the corresponding n-propylamide-n-butylboronate, and deuterium labeled [^2^H_7_]MVL was first used as an internal standard.

In 1991, Scoppola et al. (Scoppola et al. 1991) extended this approach, and quantified plasma MVA concentrations. The MVA was lactonized, extracted with [^2^H_3_]MVL and reconverted to the free acid. The resulting MVA was then converted to 3,5-bis(trifluoromethyl)benzyl ester followed by its trimethylsilyl (TMS) ether derivative. The quantification method was based on GC-CI-MS using ammonia as a reagent gas and the detection limit of MVA in plasma was 100 pg/mL. The GC-CI-MS method for the quantification of plasma MVA was subsequently improved by Ishihama et al. (Ishihama et al. 1994) and Saisho et al. (Saisho et al. 1997), and the method for the determination of urinary MVA was developed by Siavoshian et al. (Siavoshian et al. 1995). However, the GC-CI-MS methods have one disadvantage in that they required frequent cleaning of the CI ion source to maintain the high sensitivity.

To eliminate the aforementioned tedious operations in GC-CI-MS, another approach by gas chromatography-electron ionization-mass spectrometry (GC-EI-MS) was also developed. Cighetti et al. (Cighetti et al. 1981; Galli Kienle, 1984) assayed HMGCR activity by GC-EI-MS after conversion of MVL into the corresponding trimethylsilyl (TMS) ether. They used the ions at m/z 187 (M–15) for MVL-TMS and m/z 150 (M–15–CH_2_CO) for [^2^H_5_]MVL-TMS because these ions were not influenced by interfering peaks in extracts from liver microsomes. In 1989, the same group improved their original method by using GC-high-resolution (HR)-EI-MS (Del Puppo et al. 1989). This group lactonized plasma and urinary MVA into MVL using a cation exchange resin, and extracted with organic solvent after the addition of [^2^H_5_]MVL as an internal standard. The extracted MVL was then converted into the TMS ether derivative, and quantified by GC-HR-EI-MS with a mass spectral resolution of 5,000. The ions at m/z 145.0685 for MVL-TMS and m/z 150.0965 for [^2^H_5_]MVL-TMS were used for selected ion monitoring (SIM).

We also developed new assay methods to measure hepatic HMGCR activity (Honda et al. 1991) and plasma MVA concentration (Yoshida et al. 1993) by GC-HR-EI-MS. These methods made it possible to simultaneously quantify not only MVA but also 7A. Other features of these methods are described below.

- A purification procedure was developed by the serial use of commercially available solid-phase extraction cartridges, which provided high recovery and reproducibility. In brief, plasma MVA was extracted by an anion exchange Bond Elut SAX cartridge, and then eluted as MVL with 0.6 M HCl. The MVL was further purified by a reversed phase Bond Elut C18 and a normal phase Bond Elut CN cartridges. In addition, an excess benzylamine was removed by another Bond Elut CN cartridge after derivatization into mevalonylbenzylamide. The recovery of spiked MVA through the purification procedures using these cartridges was 94.1%, and the relative standard deviations between sample preparations and between measurements by this method were 5.6% and 2.8%, respectively (Yoshida et al. 1993).
- [^2^H_7_]MVL was used as an internal standard. This hepta-deuterated variant of MVL provided both good linearity of the calibration curve and easiness to distinguish between MVL peak and interfering peaks even if the MVL peak was small.
- MVL was easily converted into mevalonylbenzylamide without any catalyst under mild conditions followed by its dimethylethylsilyl (DMES) ether derivative. This amidation via MVL from MVA is a characteristic reaction for γ-hydroxyfatty acids, such as MVA, however, the free fatty acids also present in the extract did not react without catalysts. The resulting derivative gave a [M-C_2_H_5_]^+^ ion at *m/z* 380.2077 with a prominent intensity in the high mass region, which was a great advantage in the elimination of interfering peaks originating from endogenous substances in the extract by GC-EI-MS.
- The DMES ether derivative was much more stable than the TMS ether derivative.
- The MVL derivative was quantified by GC-HR-EI-MS with a mass spectral resolution of 10,000, which was also useful to eliminate peaks of unknown substances that could interfere with the monitoring.
- Trace amounts, less than 1 pg, of MVA could be detected by this method, and the lower limit of quantification in plasma sample was 180 pg/mL.
- Using these methods, it was validated that there was a highly significant correlation between the hepatic HMGCR activities and plasma concentrations of MVA in ten patients (r = 0.83, P < 0.01) (Yoshida et al. 1993).
- The GC-EI-MS method did not require frequent cleaning. This indicated that the GC-EI-MS method was suitable for clinical applications, in which it is necessary to assay a large number of samples at once.

### LC-MS and LC-MS/MS

Since the early 2000s, LC-MS or LC-MS/MS have been used more extensively than GC-MS to analyze relatively polar compounds, such as MVA or MVL, because LC-MS and LC-MS/MS do not generally require a derivatization step.

Park et al. (Park et al. 2001) and Ndong-Akoume et al. (Ndong-Akoume et al. 2002) assessed HMGCR activity by measuring MVL with LC-MS and LC-MS/MS using the positive electrospray ionization (P-ESI) mode. Plasma and urinary MVA concentrations were quantified by LC-P-ESI-MS/MS after conversion into MVL (Abrar and Martin, 2002), as well as directly by LC-negative (N)-ESI-MS/MS without lactonization (Jemal et al. 2003; Saini et al. 2006). The detection limit of MVL by the LC-MS method was 6.5 pg (Park et al. 2001), and the lower limit of quantification of plasma MVA by the LC-MS/MS methods were 200–500 pg/mL, which were similar to those obtained using GC-MS methods.

**Figure 1.**
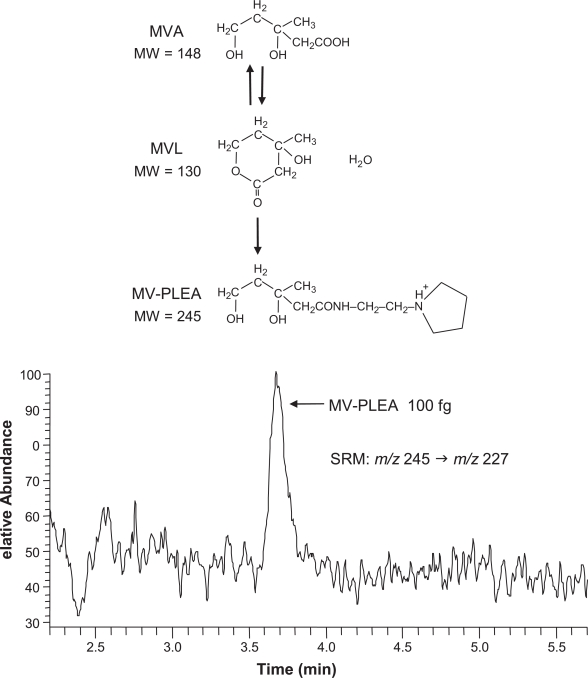
Representative chromatogram of mevalonyl-(2-pyrrolidin-1-yl-ethyl)-amide (MV-PLEA) by positive ESI-SRM at *m/z* 245 → *m/z* 227. Authentic standard of MV-PLEA (100 fg) was injected into the HPLC. LC-MS/MS conditions have been described previously (Honda et al. 2007a).

Recently, we developed a highly-sensitive method to assess HMGCR activity by LC-MS/MS (Honda et al. 2007a). In this method, MVA was extracted as MVL and its detection sensitivity was enhanced through derivatization (Fig. 1). The features of this method are described below.

- The P-ESI mode was selected to quantify MVA because the positive mode provides more abundant ions than the negative mode (Hiraoka and Kudaka, 1992).
- To select the most suitable derivative of MVA for P-ESI mode, the amidation reaction from MVA via MVL, a characteristic reaction for γ-hydroxy fatty acids such as MVA, was conducted using seven types of primary alkylamines with a tertiary amine moiety to promote protonation. Of these amide derivatives, mevaonyl-2-pyrrolidin-1-yl-ethyl)-amide (MV-PLEA) was the best derivative for the LC-P-ESI-MS/MS method.
- The detection limit of this MV-PLEA was about 30 fg (signal-to-noise ratio (S/N) = 3), indicating that this is the most sensitive method at present for the detection of MVL.
- [^2^H_7_]MVL was used as an internal standard. The recovery of spiked MVA was 94.6%, and the relative standard deviations between sample preparations and between measurements by this method were 3.2% and 1.8%, respectively.
- MV-PLEA was determined by selected reaction monitoring (SRM) using m/z 245 (M+H) as a precursor ion and m/z 227 (M+H–H_2_O) as a product ion, which almost completely eliminated the interfering peaks on the SRM chromatogram.
- Hepatic HMGCR activities in 11 normal rats were measured by both the RI and LC-P-ESI-MS/MS methods. The HMGCR activities obtained by the present method correlated well with those obtained by the conventional RI method (r = 0.93, P < 0.0001). In the RI method, [^14^C]HMG-CoA is usually used as 30 dpm/pmol = 33.3 fmol/dpm. When the standard deviation of background noise is 2 dpm, the signal would be 6 dpm when the S/N = 3. Therefore, the detection limit of the conventional RI method is calculated to be 200 fmol (S/N = 3). In comparison, the detection limit of the LC-P-ESI-MS/MS method is 240 amol (S/N = 3), ∼800 times more sensitive than that of the conventional RI method.

### HPLC

In 2005, Buffalini et al. reported a new method for the determination of HMGCR activity by HPLC (Buffalini et al. 2005). In this method, MVL produced from unlabeled HMG-CoA was extracted and quantified by HPLC with a fixed ultraviolet (UV) detector (200 nm). This method does not require very expensive equipment, such as a mass spectrometer, but the detection limit of MVL is at least 100,000 times less than that by mass spectrometry.

## The methods for the quantification of 7A

CYP7A1 activity has previously been assayed by measuring the radioactivity of 7A produced from exogenously added [^14^C]cholesterol by incubation with liver microsomes (Shefer et al. 1968). However, the extent of equilibration of exogenous labeled cholesterol with the endogenous cholesterol pool under different conditions still remains to be elucidated. To overcome this problem, several methods, i.e. a radioisotope derivative method, and GC-MS and HPLC methods, have been developed. These methods are able to measure the net amount of 7A produced from endogenous and exogenous cholesterol. Table 2 summarizes the previously reported methods for the direct determination of the mass of 7A in the liver (enzyme assay) or plasma.

### Radioisotope derivative method

This technique can measure the net amount of 7A produced from exogenous [^14^C]cholesterol and endogenous unlabeled cholesterol (Mitropoulos et al. 1972; Shefer et al. 1975). The resultant 7A was extracted, acetylated with [^3^H]acetic anhydride and purified by thin layer chromatography (TLC). The mass of 7A was calculated from the amount of radioactivity in the acetylated product based upon the specific radioactivity of the reagent.

### GC-MS

In 1974, Björkhem and Danielsson developed a method for the assay of hepatic CYP7A1 activity by GC-MS (Björkhem and Danielsson, 1974). Their method was based on stable isotope dilution-mass spectrometry using [^2^H_3_]7A as an internal standard. In this method, 7A produced from endogenous microsomal cholesterol was extracted in organic solvent, purified by TLC, converted to the TMS ether derivative, and analyzed by GC-MS. In 1981, Sanghvi et al. reported an alternative method by GC-MS in which 7A produced from microsomal cholesterol was extracted with 5α-cholestane as an internal standard by organic solvent, converted to the TMS ether derivative, and quantified by SIM (Sanghvi et al. 1981). Meanwhile, Yamashita et al. measured hepatic CYP7A1 activity by GC-SIM using 5α-cholestane-3β,7β-diol as an internal standard (Yamashita et al. 1989).

We also developed a new assay method for hepatic CYP7A1 activity by GC-HR-SIM (Honda et al. 1991). As mentioned in the previous MVA section, this method made it possible to quantify simultaneously not only 7A but also MVA. [^2^H_7_]7A was used as an internal standard and 7A was converted into its DMES ether derivative before analysis by GC-HR-MS. This DMES ether derivative was not only more stable but also much advantageous compared with the TMS ether derivative for the separation of 7A from contaminated cholesterol on GC chromatograms.

The concentration of 7A in human serum was first quantified by Björkhem et al. using GC-SIM (Björkhem et al. 1987b). They also showed that serum free (unesterified) 7A reflected hepatic CYP7A1 activities in humans. In contrast, Oda et al. quantified human serum free and esterified 7A concentrations by GC-SIM and reported that the hepatic CYP7A1 activities correlated better with the serum esterified 7A than with the free 7A (Oda et al. 1990).

In 1993, we applied our GC-HR-SIM method to the determination of human serum 7A concentrations and confirmed that there was a significant correlation (r = 0.76, p < 0.05) between serum free 7A concentrations and hepatic CYP7A1 activities in humans (Yoshida et al. 1993). However, neither the esterified 7A (r = 0.45, p > 0.05) nor the total (free + esterified) 7A concentrations (r = 0.51, p > 0.05) correlated significantly with CYP7A activities.

### HPLC

The assay method for hepatic CYP7A1 activity by HPLC was first reported by Noshiro et al. (Noshiro et al. 1985). The 7A produced from microsomal cholesterol was extracted and separated by normal phase HPLC. Although the absorption maximum of 7A was lower than 200 nm, they monitored 7A at 214 nm because there was an interference due to absorption of oxygen and/or solvent impurities at lower wavelengths.

In 1986, the same group improved the assay method by converting the produced 7A into C4 by incubating with cholesterol oxidase (Ogishima et al. 1986). Because C4 exhibits an intense absorption at 240 nm and there are fewer interfering peaks at this wavelength than at 214 nm, this improved method exhibited a more than 10-fold increase in the sensitivity compared with the previous one (Noshiro et al. 1985). In 1989, Hylemon et al. modified Ogishima’s method by using reverse-phase HPLC and adding 7β-hydroxycholesterol as an internal standard (Hylemon et al. 1989).

## The Methods for the Quantification of C4

Another plasma or serum marker for the evaluation of hepatic CYP7A1 activities is C4, which is a product of the next oxidative enzymatic reaction after CYP7A1. In fact, CYP7A1 activities correlated better with serum C4 levels compared with those of 7A irrespective of the esterification (Yoshida et al. 1994). Table 3 shows the previously described methods for the quantification of serum C4 concentrations by HPLC, GC-MS, and LC-MS/MS.

### HPLC

In 1988, Axelson et al. (Axelson et al. 1988) reported a method for the quantification of plasma C4 using normal-phase HPLC with UV detection, and demonstrated that plasma C4 concentration reflected bile acid biosynthesis in humans. In addition, they reported that there was a strong positive correlation between the plasma levels of C4 and the activities of CYP7A1 in patients treated with cholestyramine, chenodeoxycholic acid, or ursodeoxycholic acid (Axelson et al. 1991). However, their method required the addition of ^3^H-labeled 25-hydroxyvitamin D_3_ as an internal standard. On the other hand, Pettersson et al. (Pettersson and Eriksson, 1994) and Gälman et al. (Gälman et al. 2003) used unlabeled 7β-hydorxy-4-cholesten-3-one as an internal standard and analyzed C4 levels using HPLC with a reversed-phase column. The detection limits of C4 by these HPLC-UV methods were nearly 1 ng, so that at least 1 mL of plasma was required for each assay.

### GC-MS

In 1994, we developed a more sensitive method for the quantification of plasma C4 by GC-HR-MS using [^2^H_7_]C4 as an internal standard (Yoshida et al. 1994). C4 was extracted from 200 μL of plasma by a salting-out extraction, and then purified by serial solid-phase extractions. The extract was treated with O-methylhydroxylamine hydrochloride and then dimethylethylsilylated. The resulting methyloxime-DMES ether derivative was quantified by GC-HR-SIM. This method was very sensitive as well as specific, and a lower limit of detection of 1 pg was achieved.

We compared the relationships between hepatic CYP7A1 activity and plasma concentrations of C4 and free 7A in humans using our GC-HR-SIM methods (Yoshida et al. 1994). Both biomarkers correlated significantly with hepatic CYP7A1 activity (C4: r = 0.84, p < 0.001; free 7A: r = 0.73, p < 0.01), and C4 correlated better with CYP7A1 activity compared with free 7A. However, perhaps these plasma markers do not precisely reflect hepatic CYP7A1 activities in some patients with markedly changed concentrations of plasma lipoproteins. Because plasma oxysterols including C4 and 7A are transported in lipoproteins, the concentrations of oxysterols can be affected by the half-life of the lipoproteins. This hypothesis was supported by another study by ourselves (Honda et al. 2004), in which plasma C4 concentrations and hepatic CYP7A1 activities were compared in New Zealand white rabbits that were fed a high cholesterol diet and/or a bile fistula was constructed. Feeding cholesterol markedly increased and bile drainage reduced plasma cholesterol concentrations. Initially, in these models there was no correlation between plasma C4 concentrations and hepatic CYP7A1 activities (r = −0.24, p > 0.05). Cholesterol feeding was associated with downregulated CYP7A1 activities, while plasma C4 concentrations were elevated in the presence of increased plasma cholesterol levels. However, this discrepancy was overcome and a significant correlation was observed (r = 0.73, p < 0.05) by expressing C4 levels relative to cholesterol. These results suggested that plasma C4 relative to cholesterol was a better marker for hepatic CYP7A1 activity than the absolute concentration when plasma cholesterol concentrations were changed markedly.

### LC-MS/MS

**Figure 2.**
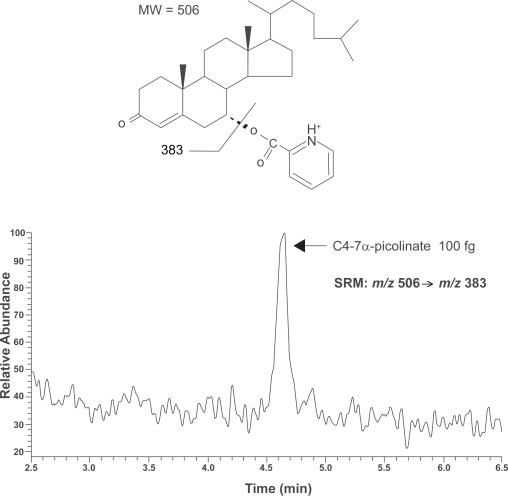
Representative chromatogram of C4-7α-picolinate by positive ESI-SRM at *m/z* 506 → *m/z* 383. Authentic standard of C4–7α-picolinate (100 fg) was injected into HPLC. LC-MS/MS conditions have been described previously (Honda et al. 2007b).

HPLC with UV detection is a more convenient method than GC-MS for the measurement of plasma C4 concentrations. However, the sensitivity is not sufficient to quantify C4 in limited amounts of human serum. Therefore, we recently developed a highly-sensitive new method by LC-MS/MS (Honda et al. 2007b). After the addition of [^2^H_7_]C4 as an internal standard, C4 was extracted from human serum (2–50 μL) by a salting-out procedure, derivatized into the picolinoyl ester (C4–7α-picolinate), and then purified using a disposable C_18_ cartridge. The resulting picolinoyl ester derivative of C4 was quantified by LC-P-ESI-MS/MS (Fig. 2). LC-MS/MS method do not always require a derivatization step. However, it is also true that the introduction of charged moieties enhances the ionization efficiency of neutral steroids in ESI and atmospheric pressure chemical ionization processes. In P-ESI mode, the picolinoyl ester of C4 exhibited an [M+H]^+^ ion at *m/z* 506 as the base peak. In the MS/MS spectrum, the [M–C_6_H_5_O_2_N]^+^ ion was observed at *m/z* 383 as the most prominent peak. The SRM was conducted using *m/z* 506 → *m/z* 383 for the C4–7α-picolinate and *m/z* 513 → *m/z* 390 for the [^2^H_7_] variant. The detection limit of the C4–7α-picolinate was 30 fg (S/N = 3), which was more than 1,000 times more sensitive than that of C4 with a conventional HPLC-UV method. The recovery of spiked C4 was 93.4%, and the relative standard deviations between sample preparations and between measurements by this method were 5.7% and 3.9%, respectively. Thus, this LC-MS/MS method is not only the most sensitive method at present for the detection of C4 but it is also highly reliable and reproducible.

## Applications to Clinical Studies

The quantification of MVA, 7A or C4 in human blood has made it possible to monitor *in vivo* cholesterol and bile acid synthesis without the need for an invasive liver biopsy. Therefore, these methods are very useful for basic or clinical time-course studies of cholesterol metabolism (Table 4).

### Diurnal cycle

In 1982, Parker et al. observed the diurnal cycle of the MVA concentrations in human plasma (Parker et al. 1982). At the peak of the cycle (between midnight and 3 a.m.), the MVA concentrations were 3–5 times greater than those at the nadir (between 9 a.m. and noon). Pappu et al. also reported that the plasma concentrations of MVA exhibited a diurnal cycle in normal subjects and patients with abetalipoproteinemia, and the highest levels were observed between midnight and 4 a.m. (Pappu and Illingworth, 1994).

On the other hand, the diurnal cycle of bile acid biosynthesis in the human liver was reported (deletion) by Duane et al. (Duane et al. 1983). They used a radioisotope technique and demonstrated for the first time that humans with an intact enterohepatic circulation exhibited a diurnal cycle of bile acid synthesis with an amplitude of ± 35%–55% around mean synthesis, and an acrophase at about 9 a.m. The same group also reported in 1988 that neither chenodeoxycholic acid nor ursodeoxycholic acid administration significantly altered the circadian rhythm of bile acid synthesis in humans (Pooler and Duane, 1988).

**Figure 3.**
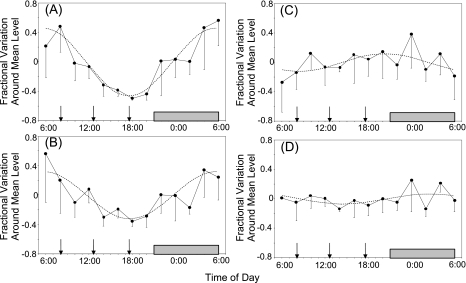
The circadian rhythm of the plasma levels of C4 and 7A in three normal volunteers. The volunteers consumed a normal hospital diet three times a day (shown by arrows), and slept from 21:00 on the first day to 6:00 on the second day (shown by the shaded box). The values are expressed as fractional variations around the mean levels (mean ± SD). Dashed lines represent the curves of best fit. (**A**) C4; (**B**) free 7A; (**C**) esterified 7A; (**D**) total 7A. Reprinted with minor modification from our previous paper (Yoshida et al. 1994), Copyright (1994), with permission from Elsevier.

**Figure 4.**
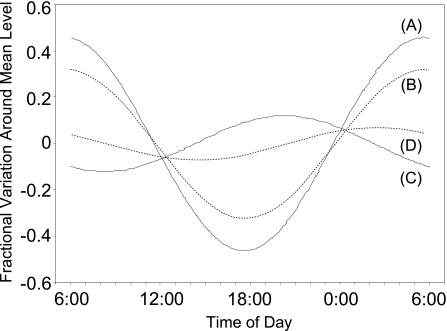
The curves of best fit for C4 and 7A. (**A**) C4, y = 0.46 cos ((2π/24)t − 1.46) (p < 0.005); (**B**) Free 7A, y = 0.32 cos ((2π/24)t − 1.48) (p < 0.005); (**C**) Esterified 7A, 0.12 cos ((2π/24)t + 0.97) (NS); (**D**) Total 7A, 0.07 cos ((2π/24)t − 0.65) (NS). Statistical significance was evaluated by a Zero-amplitude test by Nelson et al. (Nelson et al. 1979). Reprinted with minor modification from our previous paper (Yoshida et al. 1994), Copyright (1994), with permission from Elsevier.

We investigated the diurnal cycle of bile acid biosynthesis by using plasma C4 and 7A concentrations (Yoshida et al. 1994). Plasma was obtained every 2 hours from three normal volunteers and the C4 and 7A concentrations were determined using our GC-HR-MS method (Fig. 3). These levels were fitted to a cosine curve as reported in the previous studies using the isotope kinetic method (Duane et al. 1983; Pooler and Duane, 1988). The amplitudes of C4 and free 7A averaged 45% and 32%, respectively, and the acrophases of C4 and free 7A averaged 5:35 a.m. and 5:39 a.m., respectively, which was compatible with the previous results obtained using the radioisotope technique (Fig. 4). In contrast, total 7A and esterified 7A did not exhibit any significant diurnal cycle.

In 2005, Gälman et al. also reported the diurnal cycle of C4 and lathosterol, another biomarker of cholesterol biosynthesis (Gälman et al. 2005). They concluded that bile acid synthesis in humans exhibits a diurnal cycle with 2 peaks during the daytime, which is opposite from the circadian rhythm of cholesterol biosynthesis. These results were different from previous studies. Further investigations will be required to elucidate the reason for the discrepancy.

### HMGCR inhibitors

The measurement of plasma MVA concentration is very useful to evaluate the *in vivo* effects of HMGCR inhibitors. Pfohl et al. reported that the HMGCR inhibitor, simvastatin, rapidly down-regulated cholesterol biosynthesis, which was then up-regulated when the drug was withdrawn (Pfohl et al. 1998). Nozaki et al. investigated the difference in the effect of another HMGCR inhibitor, pravastatin, on cholesterol biosynthesis between the morning and the evening. They administered pravastatin to the same patients in the morning or evening, and found that morning and evening administrations of pravastatin elicited equivalent reductions in the plasma and urinary MVA concentrations (Nozaki et al. 1996). Pappu and Illingworth demonstrated that patients with familial hypercholesterolemia exhibited a diurnal pattern in plasma MVA levels similar to that reported previously in controls (Pappu and Illingworth, 2002). In addition, they reported that the administration of lovastatin in the evening reduced the nocturnal increases in MVA levels, and the administration of simvastatin completely abolished the nighttime rise. Naoumova et al. treated familial hypercholesterolemia patients with 3 different HMGCR inhibitors, pravastatin, simvastatin, and atorvastatin, and showed that the patients who responded well to statins exhibited higher basal plasma levels of MVA (Naoumova et al. 1996).

We investigated the short-term effects of pravastatin on cholesterol and bile acid biosynthesis by measuring MVA and C4 as biomarkers (Yoshida et al. 1997). Six male volunteers were administered 40 mg of pravastatin, and the plasma MVA and C4 levels were measured every 2 hours. The plasma MVA levels 2 hours after the administration of pravastatin were decreased compared with those in controls. While the decrease in MVA concentrations continued for 8 hours, the plasma C4 concentrations did not change during the initial 6 hours and then decreased 8 hours after the administration. Three-way analysis of this study indicated that the MVA level was influenced significantly by both pravastatin treatment and the time-course. In contrast, C4 level was affected significantly by both inter-individual differences and time-course, but not by pravastatin treatment. These results indicated that cholesterol biosynthesis was inhibited by pravastatin treatment, but bile acid biosynthesis was not influenced in normal subjects (Yoshida et al. 1997). Naoumova et al. treated familial hypercholesterolemia patients with atorvastatin and partial ileal bypass (Naoumova et al. 1999). Atorvastatin decreased the rate of bile acid synthesis only when bile acid synthesis was up-regulated by partial ileal bypass or bile acid sequestrants, presumably by limiting the supply of newly synthesized free cholesterol.

### Hormones

There are several reports that show the effects of hormones e.g. insulin, growth hormone and thyroid hormone, on *in vivo* cholesterol metabolism. Euglycemic hyperinsulinemia acutely decreased the circulating levels of MVA (Lala et al. 1994), which indicated that insulin could decrease cholesterol biosynthesis. Naoumova et al. also investigated the effects of hyperinsulinemia on the plasma MVA concentrations and reported that acute hyperinsulinemia decreased cholesterol biosynthesis less in the subjects with non-insulin-dependent diabetes mellitus compared with non-diabetic subjects, which suggests that the patients with non-insulin-dependent diabetes mellitus exhibit insulin resistance (Naoumova et al. 1996).

Because plasma growth hormone levels and cholesterol biosynthesis are both increased during sleep, Boyle et al. speculated that growth hormone might stimulate *de novo* cholesterol biosynthesis (Boyle et al. 1992). However, the peak nocturnal and fasting MVA concentrations did not correlate with the growth hormone levels, and they concluded that nocturnal growth hormone secretion was not related to the stimulation of cholesterol production during sleep.

Patients with hypothyroidism exhibit hypercholesterolemia, while those with hyperthyroidism exhibit hypocholesterolemia. Sauter et al. measured serum C4 concentrations before and after treatment for hypo- and hyperthyroidism and showed that in humans, thyroid hormones influenced the serum cholesterol concentrations by mechanisms other than through modification of the CYP7A1 activity (Sauter et al. 1997).

### Bile acids

Bile acids, particularly chenodeoxycholic acid (CDCA) and deoxycholic acid (DCA), are physiological ligands for the farnesoid X receptor (FXR, NR1H4). They inhibit bile acid biosynthesis through activation of this nuclear receptor. In fact, Einarsson et al. reported that the treatment of healthy subjects with CDCA or DCA reduced the serum concentrations of C4 (Einarsson et al. 2001). They also found that CDCA reduced cholesterol biosynthesis while DCA did not when they evaluated *in vivo* cholesterol biosynthesis by measuring the serum 7-dehydrocholesterol concentrations. In contrast, UDCA treatment for 40 days did not affect cholesterol synthesis, as evaluated by urinary excretion of MVA, but the same treatment significantly increased bile acid biosynthesis determined by serum C4 concentrations (Sauter et al. 2004).

### Hepatobiliary diseases

Cholesterol gallstone disease is caused by abnormal cholesterol and bile acid metabolism. The formation of cholesterol supersaturated bile is one of the important factors in the pathogenesis of this disease. Shoda et al. proposed an estimated biliary cholesterol saturation index (CSI)_E_ = 1[MVL] + 0.7[C4] that was calculated by multivariate linear regression analysis using the plasma MVA and C4 concentrations of patients with hyperlipoproteinemia and demonstrated that this convenient calculation of (CSI)_E_ corresponded well to actual biliary CSI (Shoda et al. 1997). However, the hypersecretion of biliary cholesterol in patients with gallstones does not seem to be due to increased hepatic synthesis of cholesterol or decreased catabolism of cholesterol to bile acids. This could be because the plasma levels of lathosterol were not significantly different between gallstone subjects and controls and the C4 levels were about 40% higher in the gallstone subjects compared with the controls (Muhrbeck et al. 1997). The increased bile acid biosynthesis determined by the plasma C4 levels, corrected for plasma cholesterol, was also reported in gallstone subjects and gallstone high-risk Mapuche Indians (Gälman et al. 2004).

Conversely, some hepatobiliary diseases affect cholesterol and bile acid metabolism. In patients with liver cirrhosis (LC), the blood cholesterol levels are relatively preserved, despite other markers, including the serum albumin levels, show liver dysfunction. We studied the association between hepatic cholesterogenesis and bile acid synthesis in hepatocellular impairment using the plasma levels of MVA and C4 (Yoshida et al. 1999). There were no significant differences in the plasma MVA levels between chronic hepatitis (CH), LC and control groups. In contrast, plasma C4 levels were significantly lower in LC compared with the CH and control groups. Although the MVA levels did not correlate with the Child-Pugh’s score, which reflects the severity of liver damage (Albers et al. 1989), there was a significant correlation between the C4 level and Child-Pugh’s score. In addition, plasma C4 levels in the control subjects correlated positively with the MVA levels, but there was no significant correlation between these biomarkers in CH and LC patients. Therefore, it was concluded that in the patients with chronic liver disease, there was a tendency for hepatic cholesterogenesis to be sustained in the face of hepatocellular dysfunction, while bile acid synthesis declined in parallel with the severity of impairment.

## Perspectives

Biological specimens contain many types of organic acids and sterols. While fatty acids and cholesterol are relatively abundant compounds, MVA and oxysterols (7A and C4) are minor components. To quantify the concentrations of such minor components, stable isotope dilution mass spectrometry (GC-MS or LC-MS/MS) is an ideal method because of its high sensitivity, specificity and accuracy.

Recently, LC-MS/MS has come to be used more readily than GC-MS. Because MS/MS is more specific than MS, sample preparation process for the elimination of interfering materials can be simplified. In addition, LC-MS/MS does not require a derivatization step, which is also advantageous for high-throughput analyses. However, simple and rapid procedures do not always produce good results for the microanalysis of biological samples. A careful sample purification can increase the sensitivity of an analyte by reducing matrix effect (Jemal et al. 2003). Derivatization is useful not only to increase the sensitivity by enhancing the ionization efficiency but also to give a prominent ion in the high mass region, which makes it possible to avoid interfering peaks and to increase the specificity. A thorough chromatographic separation is also important to distinguish between similar biological compounds, e.g. hydroxycholesterols that have the same molecular weight and a virtually identical MS/MS spectrum. Thus, the importance of basic analytical techniques, i.e. sample purification, derivatization and chromatographic separation will not be denied even if the performance of mass spectrometer is improved further.

In conclusion, the methods for the quantification of key intermediates in cholesterol and bile acid biosynthetic pathways using stable isotope dilution mass spectrometry exhibit superior accuracy and sensitivity. By using this technique, the MVA and oxysterols in blood were established as biomarkers for cholesterol and bile acid biosynthesis. The use of these biomarkers has made it possible to monitor *in vivo* cholesterol and bile acid synthesis without the need for invasive liver biopsy, which is very useful for basic or clinical studies of cholesterol metabolism in humans.

## Figures and Tables

**Table 1.** Methods for quantification of MVA in biological samples.

**Author**	**Year**	**Method (ionization mode)**	**Derivatization**	**Lower limit of detection**	**Intra-assay variation**	**Inter-assay variation**	**Recovery**	**Application**
Hagenfeldt et al.	1972	GC-MS (P-EI)	MVL	NA	NA	6.2%	87% ± 4%	blood
Miyazaki et al.	1978	GC-MS (P-CI)	MVL-PABB	NA	NA	NA	NA	liver
Popjak et al.	1979	radioenzymatic assay	5-[^32^P]phospho-MVA	150–300 pg	NA	NA	100%	plasma
Cighetti et al.	1981	GC-MS (P-EI)	MVL-TMS	NA	1.5%	6.1%	NA	liver
Del Puppo et al.	1989	GC-HR-MS (P-EI)	MVL-TMS	NA	6.5%	NA	101% ± 4%	plasma urine
Honda et al.	1991	GC-HR-MS (P-EI)	MVL-B-DMES	800 fg	4.9%	7.8%	96%–100%	liver
Scoppola et al.	1991	GC-MS (N-CI)	MVA-TFB-TMS	10 pg	5.1%	7.7%	NA	plasma
Yoshida et al.	1993	GC-HR-MS (P-EI)	MVL-B-DMES	NA	2.8%	5.6%	91%–96%	plasma
Ishihama et al.	1994	GC-MS (P-CI)	MVL	NA	2.2%	4.5%	101%–103%	plasma
Siavoshian et al.	1995	GC-MS (P-CI)	MVL-TMS	NA	4.0%	8.0%	70% ± 2%	urine
Saisho et al.	1997	GC-MS (N-CI)	MVA-PFB-CB	NA	2.0%	7.5%	100%–107%	plasma
Hiramatsu et al.	1998	enzyme immunoassay	MVA	195 pg	3.4%	5.2%	102% ± 7%	urine
Woollen et al.	2001	GC-MS (P-EI)	MVL	NA	<13.7%	<9.8%	82%–110%	urine
Park et al.	2001	LC-MS (P-ESI)	MVL	6.5 pg	4.1%	9.4%	95% ± 4%	liver
Ndong-Akoume et al.	2002	LC-MS/MS (P-ESI)	MVL	NA	<1.0%	NA	98%–99%	liver
Abrar et al.	2002	LC-MS/MS (P-ESI)	MVL	NA	4.1%–15%	13%–16%	89%–114%	plasma
Jemal et al.	2003	LC-MS/MS (N-ESI)	MVA	NA	<4.5%	<3.3%	98%–103%	plasma urine
Buffalini et al.	2005	HPLC-UV	MVL	741 ng	NA	<3.0%	97%–103%	liver
Saini et al.	2006	LC-MS/MS (N-ESI)	MVA	NA	1%–17%	3%–12%	99%–108%	plasma
Honda et al.	2007	LC-MS/MS (P-ESI)	MV-PLEA	31 fg	1.8%	3.2%	93%–96%	liver

**Abbreviations:** P-EI: positive electron ionization; NA: not available; P-CI: positive chemical ionization; N-CI: negative chemical ionization; MVL-PABB: MVL n-propylamide-n-butylboronate; MVL-TMS: trimethylsilyl ether of MVL; GC-HR-MS: high-resolution GC-MS; MVL-B-DMES: dimethylethylsilyl ether of mevalonylbenzylamide; MVA-TFB-TMS: trimethylsilyl ether of bis(trifluoromethyl)benzyl ester of MVA; MVA-PFB-CB: cyclic boronate-pentafluorobenzyl ester of MVA; P-ESI: positive electrospray ionization; N-ESI: negative electrospray ionization; HPLC-UV: high-performance liquid chromatography equipped with an ultraviolet detector; MV-PLEA: MV-(2-pyrrolidin-1-yl-ethyl)-amide.

**Table 2.** Methods for quantification of 7A in biological samples.

**Author**	**Year**	**Method (ionization mode)**	**Derivatization**	**Lower limit of detection**	**Intra-assay variation**	**Inter-assay variation**	**Recovery**	**Application**
Mitropoulos et al.	1972	radioisotope derivative method	acetylated 7A	NA	NA	NA	NA	liver
Björkhem et al.	1974	GC-MS (P-EI)	7A-TMS	NA	NA	2.2%	95%	liver
Sanghvi et al.	1981	GC-MS (P-EI)	7A-TMS	50 pg	3.5%	2.3%–4.7%	NA	liver
Noshiro et al.	1985	NP-HPLC-UV	7A	NA	NA	NA	385%	liver
Ogishima et al.	1986	NP-HPLC-UV	C4	NA	NA	NA	NA	liver
Björkhem et al.	1987	GC-MS (P-EI)	7A-TMS	1–2 ng/mL	NA	4%–8%	105%	serum
Hylemon et al.	1989	RP-HPLC-UV	C4	8 ng	NA	NA	NA	liver
Yamashita et al.	1989	GC-MS (P-EI)	7A-TMS	NA	3.8%	4.6%	92%–99%	liver
Oda et al.	1990	GC-MS (P-EI)	7A-TMS	NA	NA	3%	97%–109%	serum
Honda et al.	1991	GC-HR-MS (P-EI)	7A-DMES	1.6 pg	7.9%	7.0%	94%–102%	liver
Yoshida et al.	1993	GC-HR-MS (P-EI)	7A-DMES	NA	4.2%	2.6%	93%–95%	serum

**Abbreviations:** NA: not available; P-EI: positive electron ionization; 7A-TMS: trimethylsilyl ether of 7α-hydroxycholesterol; HPLC-UV: high-performance liquid chromatography equipped with an ultraviolet detector; NP: normal-phase; C4: 7α-hydroxy-4-cholesten-3-one; RP: reversed-phase; GC-HR-MS: high-resolution GC-MS; 7A-DMES: dimethylethylsilyl ether of 7α-hydroxycholesterol

**Table 3.** Methods for quantification of serum C4 concentration.

**Author**	**Year**	**Method (ionization mode)**	**Derivatization**	**Lower limit of detection**	**Intra-assay variation**	**Inter-assay variation**	**Recovery**	**Application**
Axelson et al.	1988	NP-HPLC-UV	C4	0.5–1.5 ng/mL	NA	5%	82%–106%	plasma
Pettersson et al.	1994	RP-HPLC-UV	C4	3 ng	3.2%	3.8%	96%–105%	serum
Yoshida et al.	1994	GC-HR-MS (P-EI)	C4-MO-DMES	1 pg	2.54%	5.16%	94%–100%	plasma
Gälman et al.	2003	RP-HPLC-UV	C4	500 pg	4.4%	5.6%	NA	blood
Honda et al.	2007	LC-MS/MS (P-ESI)	C4-picolinate	30 fg	3.9%	5.7%	92%–94%	serum

**Abbreviations:** HPLC-UV: high-performance liquid chromatography equipped with an ultraviolet detector; NA: not available; NP: normal-phase; RP: reversed-phase; GC-HR-MS: high-resolution GC-MS; P-EI: positive electron ionization; C4-MO-DMES: methyloxime dimethylethylsilyl ether of C4; P-ESI: positive electrospray ionization.

**Table 4.** Applications of MVA and C4 as biomarkers for cholesterol and bile acid biosynthesis.

	**MVA**	**C4**	**References**
HMGCR inhibitors	decrease	–	(Nozaki, 1996; Naoumova, 1996; Yoshida, 1997; Naoumova, 1997; Pfohl, 1998; O’Neill, 2001; Pappu, 2002)
	–	no effect	(Yoshida, 1997; Naoumova, 1999; O’Neill, 2001)
HMGCR inhibitors with partial ileal resection	decrease	decrease	(Naoumova, 1999)
Insulin	decrease	–	(Lala, 1994; Scoppola, 1995; Naoumova, 1996)
Growth hormone	no effect	–	(Boyle, 1992)
	–	no effect	(Leonsson, 1999; Lind, 2004)
Thyroid hormone	–	no effect	(Sauter, 1997)
CDCA	–	decrease	(Einarsson, 2001)
DCA	–	decrease	(Einarsson, 2001)
UDCA	–	increase	(Sauter, 2004)
Rifampin	–	increase	(Lutjohann, 2004)
Gallstone	–	increase	(Muhrbeck, 1997; Gälman, 2004)
Liver cirrhosis	no change	decrease	(Yoshida, 1999)
Diarrhea	–	increase	(Eusufzai, 1993; Sauter, 1999)

## References

[b1-aci-3-45] Abrar M Martin PD 2002 Validation and application of an assay for the determination of mevalonic acid in human plasma by liquid chromatography tandem mass spectrometry J Chromatogr B 773 103 11 10.1016/s1570-0232(02)00131-9 12031835

[b2-aci-3-45] Albers I Hartmann H Bircher J 1989 Superiority of the Child-Pugh classification to quantitative liver function tests for assessing prognosis of liver cirrhosis Scand J Gastroenterol 24 269 76 2734585 10.3109/00365528909093045

[b3-aci-3-45] Axelson M Aly A Sjövall J 1988 Levels of 7α-hydroxy-4-cholesten-3-one in plasma reflect rates of bile acid synthesis in man FEBS Lett 239 324 8 3181435 10.1016/0014-5793(88)80944-x

[b4-aci-3-45] Axelson M Björkhem I Reihnér E 1991 The plasma level of 7α-hydroxy-4-cholesten-3-one reflects the activity of hepatic cholesterol 7α-hydroxylase in man FEBS Lett 284 216 8 2060639 10.1016/0014-5793(91)80688-y

[b5-aci-3-45] Björkhem I Danielsson H 1974 Assay of liver microsomal cholesterol 7α-hydroxylase using deuterated carrier and gas chromatography-mass spectrometry Anal Biochem 59 508 16 4838781 10.1016/0003-2697(74)90304-2

[b6-aci-3-45] Björkhem I Miettinen T Reihnér E 1987a Correlation between serum levels of some cholesterol precursors and activity of HMG-CoA reductase in human liver J Lipid Res 28 1137 43 3681138

[b7-aci-3-45] Björkhem I Reihnér E Angelin B 1987b On the possible use of the serum level of 7α-hydroxycholesterol as a marker for increased activity of the cholesterol 7α-hydroxylase in humans J Lipid Res 28 889 94 3668387

[b8-aci-3-45] Boyle PJ Avogaro A Smith L 1992 Role of GH in regulating nocturnal rates of lipolysis and plasma mevalonate levels in normal and diabetic humans Am J Physiol 263 E168 72 1636694 10.1152/ajpendo.1992.263.1.E168

[b9-aci-3-45] Buffalini M Pierleoni R Guidi C 2005 Novel and simple high-performance liquid chromatographic method for determination of 3-hydroxy-3-methylglutaryl-coenzyme A reductase activity J Chromatogr B 819 307 13 10.1016/j.jchromb.2005.02.017 15833295

[b10-aci-3-45] Cighetti G Santaniello E Galli G 1981 Evaluation of 3-hydroxy-3-methylglutaryl-CoA reductase activity by multiple-selected ion monitoring Anal Biochem 110 153 8 7212258 10.1016/0003-2697(81)90128-7

[b11-aci-3-45] Del Puppo M Cighetti G Galli Kienle M 1989 Measurement of mevalonate in human plasma and urine by multiple selected ion monitoring Biomed Environ Mass Spectrom 18 174 6 2713546 10.1002/bms.1200180305

[b12-aci-3-45] Dietschy JM Brown MS 1974 Effect of alterations of the specific activity of the intracellular acetyl CoA pool on apparent rates of hepatic cholesterogenesis J Lipid Res 15 508 16 4413018

[b13-aci-3-45] Duane WC Levitt DG Mueller SM 1983 Regulation of bile acid synthesis in man. Presence of a diurnal rhythm J Clin Invest 72 1930 6 6417166 10.1172/JCI111157 PMC437033

[b14-aci-3-45] Duane WC Javitt NB 1999 27-hydroxycholesterol: production rates in normal human subjects J Lipid Res 40 1194 9 10393204

[b15-aci-3-45] Einarsson C Hillebrant CG Axelson M 2001 Effects of treatment with deoxycholic acid and chenodeoxycholic acid on the hepatic synthesis of cholesterol and bile acids in healthy subjects Hepatology 33 1189 93 11343248 10.1053/jhep.2001.23790

[b16-aci-3-45] Eusufzai S Axelson M Angelin B 1993 Serum 7α-hydroxy-4-cholesten-3-one concentrations in the evaluation of bile acid malabsorption in patients with diarrhoea: correlation to SeHCAT test Gut 34 698 701 8504974 10.1136/gut.34.5.698 PMC1374193

[b17-aci-3-45] Everson GT 1992 Bile acid metabolism and its role in human cholesterol balance Semin Liver Dis 12 420 8 1334576 10.1055/s-2008-1040411

[b18-aci-3-45] Galli Kienle M Galli G Bosisio E 1984 Evaluation of enzyme activities by gas chromatography-mass spectrometry: HMGCoA reductase and cholesterol 7α-hydroxylase J Chromatogr 289 267 76 6736156 10.1016/s0021-9673(00)95094-5

[b19-aci-3-45] Gälman C Arvidsson I Angelin B 2003 Monitoring hepatic cholesterol 7α-hydroxylase activity by assay of the stable bile acid intermediate 7α-hydroxy-4-cholesten-3-one in peripheral blood J Lipid Res 44 859 66 12562858 10.1194/jlr.D200043-JLR200

[b20-aci-3-45] Gälman C Miquel JF Perez RM 2004 Bile acid synthesis is increased in Chilean Hispanics with gallstones and in gallstone high-risk Mapuche Indians Gastroenterology 126 741 8 14988828 10.1053/j.gastro.2003.12.009

[b21-aci-3-45] Gälman C Angelin B Rudling M 2005 Bile acid synthesis in humans has a rapid diurnal variation that is asynchronous with cholesterol synthesis Gastroenterology 129 1445 53 16285946 10.1053/j.gastro.2005.09.009

[b22-aci-3-45] Goldfarb S Pitot HC 1971 Improved assay of 3-hydroxy-3-methylglutaryl coenzyme A reductase J Lipid Res 12 512 5 5164097

[b23-aci-3-45] Hagenfeldt L Hellström K 1972 Blood concentration and turnover of circulating mevalonate in the rat Life Sci 11 669 76 10.1016/0024-3205(72)90016-1 4656875

[b24-aci-3-45] Hahn C Reichel C von Bergmann K 1995 Serum concentration of 7α-hydroxycholesterol as an indicator of bile acid synthesis in humans J Lipid Res 36 2059 66 8558093

[b25-aci-3-45] Hiramatsu M Hayashi A Hidaka H 1998 Enzyme immunoassay of urinary mevalonic acid and its clinical application Clin Chem 44 2152 7 9761249

[b26-aci-3-45] Hiraoka K Kudaka I 1992 Nagative-mode electrospray-mass spectrometry using nonaqueous solvents Rapid Commun Mass Spectrom 6 265 8

[b27-aci-3-45] Honda A Shoda J Tanaka N 1991 Simultaneous assay of the activities of two key enzymes in cholesterol metabolism by gas chromatography-mass spectrometry J Chromatogr 565 53 66 1874904 10.1016/0378-4347(91)80370-r

[b28-aci-3-45] Honda A Yoshida T Xu G 2004 Significance of plasma 7α-hydroxy-4-cholesten-3-one and 27-hydroxycholesterol concentrations as markers for hepatic bile acid synthesis in cholesterol-fed rabbits Metabolism 53 42 8 14681840 10.1016/j.metabol.2003.07.018

[b29-aci-3-45] Honda A Mizokami Y Matsuzaki Y 2007a Highly-sensitive assay of HMG-CoA reductase activity by LC-ESI-MS/MS J Lipid Res 48 1212 20 17272831 10.1194/jlr.D600049-JLR200

[b30-aci-3-45] Honda A Yamashita K Numazawa M 2007b Highly-sensitive quantification of 7α-hydroxy-4-cholesten-3-one in human serum by LC-ESI-MS/MS J Lipid Res 48 458 64 17093295 10.1194/jlr.D600032-JLR200

[b31-aci-3-45] Hylemon PB Studer EJ Pandak WM 1989 Simultaneous measurement of cholesterol 7α-hydroxylase activity by reverse-phase high-performance liquid chromatography using both endogenous and exogenous [4–14C]cholesterol as substrate Anal Biochem 182 212 6 2610336 10.1016/0003-2697(89)90581-2

[b32-aci-3-45] Ishihama Y Mano N Oda Y 1994 Simple and sensitive quantitation method for mevalonic acid in plasma using gas chromatography/mass spectrometry Rapid Commun Mass Spectrom 8 377 80 8025334 10.1002/rcm.1290080507

[b33-aci-3-45] Jemal M Schuster A Whigan DB 2003 Liquid chromatography/tandem mass spectrometry methods for quantitation of mevalonic acid in human plasma and urine: method validation, demonstration of using a surrogate analyte, and demonstration of unacceptable matrix effect in spite of use of a stable isotope analog internal standard Rapid Commun Mass Spectrom 17 1723 34 12872277 10.1002/rcm.1112

[b34-aci-3-45] Kempen HJM Glatz JFC Leuven JAG 1988 Serum lathosterol concentration is an indicator of whole-body cholesterol synthesis in humans J Lipid Res 29 1149 55 3183524

[b35-aci-3-45] Lala A Scoppola A Ricci A 1994 The effects of insulin on plasma mevalonate concentrations in man Ann Nutr Metab 38 257 62 7710260 10.1159/000177819

[b36-aci-3-45] Leonsson M Oscarsson J Bosaeus I 1999 Growth hormone (GH) therapy in GH-deficient adults influences the response to a dietary load of cholesterol and saturated fat in terms of cholesterol synthesis, but not serum low density lipoprotein cholesterol levels J Clin Endocrinol Metab 84 1296 303 10199770 10.1210/jcem.84.4.5611

[b37-aci-3-45] Lind S Rudling M Ericsson S 2004 Growth hormone induces low-density lipoprotein clearance but not bile acid synthesis in humans Arterioscler Thromb Vasc Biol 24 349 56 14656733 10.1161/01.ATV.0000110657.67317.90

[b38-aci-3-45] Lutjohann D Hahn C Prange W 2004 Influence of rifampin on serum markers of cholesterol and bile acid synthesis in men Int J Clin Pharmacol Ther 42 307 13 15222722 10.5414/cpp42307

[b39-aci-3-45] Mitropoulos KA Balasubramaniam S 1972 Cholesterol 7α-hydroxylase in rat liver microsomal preparations Biochem J 128 1 9 4404423 10.1042/bj1280001 PMC1173563

[b40-aci-3-45] Miyazaki H Koyama M Hashimoto M 1978 Assay for 3-hydroxy-3-methylglutaryl coenzyme A reductase by selected ion monitoring In Stable Isotopes: Proceedings of the Third International Conference Oak Brook, IL May 23–26, 1978 Klein ER Klein PD 267 73

[b41-aci-3-45] Muhrbeck O Wang FH Björkhem I 1997 Circulating markers for biosynthesis of cholesterol and bile acids are not depressed in asymptomatic gallstone subjects J Hepatol 27 150 5 9252089 10.1016/s0168-8278(97)80295-4

[b42-aci-3-45] Naoumova RP Cummings MH Watts GF 1996 Acute hyperinsulinaemia decreases cholesterol synthesis less in subjects with non-insulin-dependent diabetes mellitus than in non-diabetic subjects Eur J Clin Invest 26 332 40 8732493 10.1046/j.1365-2362.1996.138285.x

[b43-aci-3-45] Naoumova RP Marais AD Mountney J 1996 Plasma mevalonic acid, an index of cholesterol synthesis in vivo, and responsiveness to HMG-CoA reductase inhibitors in familial hypercholesterolaemia Atherosclerosis 119 203 13 8808497 10.1016/0021-9150(95)05649-1

[b44-aci-3-45] Naoumova RP Dunn S Rallidis L 1997 Prolonged inhibition of cholesterol synthesis explains the efficacy of atorvastatin J Lipid Res 38 1496 500 9254075

[b45-aci-3-45] Naoumova RP O’Neill FH Dunn S 1999 Effect of inhibiting HMG-CoA reductase on 7α-hydroxy-4-cholesten-3-one, a marker of bile acid synthesis: contrasting findings in patients with and without prior up-regulation of the latter pathway Eur J Clin Invest 29 404 12 10354197 10.1046/j.1365-2362.1999.00475.x

[b46-aci-3-45] Ndong-Akoume MY Mignault D Perwaiz S 2002 Simultaneous evaluation of HMG-CoA reductase and cholesterol 7α-hydroxylase activities by electrospray tandem MS Lipids 37 1101 7 12558061 10.1007/s11745-002-1006-z

[b47-aci-3-45] Nelson W Tong YL Lee JK 1979 Methods for cosinorrhythmometry Chronobiologia 6 305 23 548245

[b48-aci-3-45] Noshiro M Ishida H Hayashi S 1985 Assays for cholesterol 7α-hydroxylase and 12α-hydroxylase using high performance liquid chromatography Steroids 45 539 49 3939046 10.1016/0039-128x(85)90018-2

[b49-aci-3-45] Nozaki S Nakagawa T Nakata A 1996 Effects of pravastatin on plasma and urinary mevalonate concentrations in subjects with familial hypercholesterolaemia: a comparison of morning and evening administration Eur J Clin Pharmacol 49 361 4 8866629 10.1007/BF00203778

[b50-aci-3-45] Oda H Yamashita H Kosahara K 1990 Esterified and total 7α-hydroxycholesterol in human serum as an indicator for hepatic bile acid synthesis J Lipid Res 31 2209 18 2090715

[b51-aci-3-45] Ogishima T Okuda K 1986 An improved method for assay of cholesterol 7α-hydroxylase activity Anal Biochem 158 228 32 3799966 10.1016/0003-2697(86)90613-5

[b52-aci-3-45] O’Neill FH Patel DD Knight BL 2001 Determinants of variable response to statin treatment in patients with refractory familial hypercholesterolemia Arterioscler Thromb Vasc Biol 21 832 7 11348882 10.1161/01.atv.21.5.832

[b53-aci-3-45] Pappu AS Illingworth DR 1994 Diurnal variations in the plasma concentrations of mevalonic acid in patients with abetalipoproteinaemia Eur J Clin Invest 24 698 702 7851471 10.1111/j.1365-2362.1994.tb01063.x

[b54-aci-3-45] Pappu AS Illingworth DR 2002 The effects of lovastatin and simvastatin on the diurnal periodicity of plasma mevalonate concentrations in patients with heterozygous familial hypercholesterolemia Atherosclerosis 165 137 44 12208479 10.1016/s0021-9150(02)00192-2

[b55-aci-3-45] Park EJ Lee D Shin YG 2001 Analysis of 3-hydroxy-3-methylglutaryl-coenzyme A reductase inhibitors using liquid chromatography-electrospray mass spectrometry J Chromatogr B Biomed Sci Appl 754 327 32 11339276 10.1016/s0378-4347(00)00620-4

[b56-aci-3-45] Parker TS McNamara DJ Brown C 1982 Mevalonic acid in human plasma: relationship of concentration and circadian rhythm to cholesterol synthesis rates in man Proc Natl Acad Sci USA 79 3037 41 6953446 10.1073/pnas.79.9.3037 PMC346344

[b57-aci-3-45] Parker TS McNamara DJ Brown CD 1984 Plasma mevalonate as a measure of cholesterol synthesis in man J Clin Invest 74 795 804 6565710 10.1172/JCI111495 PMC425233

[b58-aci-3-45] Pettersson L Eriksson CG 1994 Reversed-phase high-performance liquid chromatographic determination of 7α-hydroxy-4-cholesten-3-one in human serum J Chromatogr B Biomed Appl 657 31 6 7952081 10.1016/0378-4347(94)80066-9

[b59-aci-3-45] Pfohl M Naoumova RP Kim KD 1998 Use of cholesterol precursors to assess changes in cholesterol synthesis under non-steady-state conditions Eur J Clin Invest 28 491 6 9693942 10.1046/j.1365-2362.1998.00321.x

[b60-aci-3-45] Pooler PA Duane WC 1988 Effects of bile acid administration on bile acid synthesis and its circadian rhythm in man Hepatology 8 1140 6 3138171 10.1002/hep.1840080530

[b61-aci-3-45] Popjak G Boehm G Parker TS 1979 Determination of mevalonate in blood plasma in man and rat. Mevalonate “tolerance” tests in man J Lipid Res 20 716 28 226640

[b62-aci-3-45] Saini GS Wani TA Gautam A 2006 Validation of LC-MS/MS method for quantification of mevalonic acid in human plasma and an approach to differentiate recovery and matrix effect J Lipid Res 47 2340 5 16861623 10.1194/jlr.D600018-JLR200

[b63-aci-3-45] Sanghvi A Grassi E Bartman C 1981 Measurement of cholesterol 7α-hydroxylase activity with selected ion monitoring J Lipid Res 22 720 4 7276747

[b64-aci-3-45] Saisho Y Kuroda T Umeda T 1997 A sensitive and selective method for the determination of mevalonic acid in dog plasma by gas chromatography/negative ion chemical ionization-mass spectrometry J Pharm Biomed Anal 15 1223 30 9226547 10.1016/s0731-7085(96)02012-2

[b65-aci-3-45] Sauter G Berr F Beuers U 1996 Serum concentrations of 7α-hydroxy-4-cholesten-3-one reflect bile acid synthesis in humans Hepatology 24 123 6 8707250 10.1053/jhep.1996.v24.pm0008707250

[b66-aci-3-45] Sauter G Weiss M Hoermann R 1997 Cholesterol 7α-hydroxylase activity in hypothyroidism and hyperthyroidism in humans Horm Metab Res 29 176 9 9178027 10.1055/s-2007-979016

[b67-aci-3-45] Sauter GH Munzing W von Ritter C 1999 Bile acid malabsorption as a cause of chronic diarrhea: diagnostic value of 7α-hydroxy-4-cholesten-3-one in serum Dig Dis Sci 44 14 9 9952217 10.1023/a:1026681512303

[b68-aci-3-45] Sauter GH Thiessen K Parhofer KG 2004 Effects of ursodeoxycholic acid on synthesis of cholesterol and bile acids in healthy subjects Digestion 70 79 83 15375335 10.1159/000080925

[b69-aci-3-45] Schwarz M Lund EG Setchell KD 1996 Disruption of cholesterol 7α-hydroxylase gene in mice. II Bile acid deficiency is overcome by induction of oxysterol 7α-hydroxylase J Biol Chem 271 18024 31 8663430 10.1074/jbc.271.30.18024 PMC4451191

[b70-aci-3-45] Scoppola A Maher VM Thompson GR 1991 Quantitation of plasma mevalonic acid using gas chromatography-electron capture mass spectrometry J Lipid Res 32 1057 60 1940620

[b71-aci-3-45] Scoppola A Testa G Frontoni S 1995 Effects of insulin on cholesterol synthesis in type II diabetes patients Diabetes Care 18 1362 9 8721938 10.2337/diacare.18.10.1362

[b72-aci-3-45] Shapiro DJ Imblum RL Rodwell VW 1969 Thin-layer chromatographic assay for HMG-CoA reductase and mevalomic acid Anal Biochem 31 383 90 5393333 10.1016/0003-2697(69)90279-6

[b73-aci-3-45] Shefer S Hauser S Mosbach EH 1968 7α-Hydroxylation of cholesterol by rat liver microsomes J Lipid Res 9 328 33 5650927

[b74-aci-3-45] Shefer S Hauser S Lapar V 1972 HMG CoA reductase of intestinal mucosa and liver of the rat J Lipid Res 13 402 12 5025470

[b75-aci-3-45] Shefer S Nicolau G Mosbach EH 1975 Isotope derivative assay of microsomal cholesterol 7α-hydroxylase J Lipid Res 16 92 6 1127356

[b76-aci-3-45] Shoda J Tanaka N He BF 1993 Alterations of bile acid composition in gallstones, bile, and liver of patients with hepatolithiasis, and their etiological significance Dig Dis Sci 38 2130 41 8223090 10.1007/BF01297095

[b77-aci-3-45] Shoda J Miyamoto J Kano M 1997 Simultaneous determination of plasma mevalonate and 7α-hydroxy-4-cholesten-3-one levels in hyperlipoproteinemia: convenient indices for estimating hepatic defects of cholesterol and bile acid syntheses and biliary cholesterol supersaturation Hepatology 25 18 26 8985259 10.1053/jhep.1997.v25.pm0008985259

[b78-aci-3-45] Siavoshian S Simoneau C Maugeais P 1995 Measurement of mevalonic acid in human urine by bench top gas chromatography-mass spectrometry Clin Chim Acta 243 129 36 8747489 10.1016/0009-8981(95)06162-2

[b79-aci-3-45] Vlahcevic ZR Pandak WM Heuman DM 1992 Function and regulation of hydroxylases involved in the bile acid biosynthesis pathways Semin Liver Dis 12 403 19 1465624 10.1055/s-2008-1040410

[b80-aci-3-45] Vlahcevic ZR Stravitz RT Heuman DM 1997 Quantitative estimations of the contribution of different bile acid pathways to total bile acid synthesis in the rat Gastroenterology 113 1949 57 9394735 10.1016/s0016-5085(97)70015-5

[b81-aci-3-45] Woollen BH Holme PC Northway WJ 2001 Determination of mevalonic acid in human urine as mevalonic acid lactone by gas chromatography-mass spectrometry J Chromatogr 760 179 84 10.1016/s0378-4347(01)00247-x 11522061

[b82-aci-3-45] Yamashita H Kuroki S Nakayama F 1989 Assay of cholesterol 7α-hydroxylase utilizing a silica cartridge column and 5α-cholestane-3β,7β-diol as an internal standard J Chromatogr 496 255 68 2613831

[b83-aci-3-45] Yoshida T Honda A Tanaka N 1993 Simultaneous determination of mevalonate and 7α-hydroxycholesterol in human plasma by gas chromatography-mass spectrometry as indices of cholesterol and bile acid biosynthesis J Chromatogr 613 185 93 8491805 10.1016/0378-4347(93)80133-o

[b84-aci-3-45] Yoshida T Honda A Tanaka N 1994 Determination of 7α-hydroxy-4-cholesten-3-one level in plasma using isotope-dilution mass spectrometry and monitoring its circadian rhythm in human as an index of bile acid biosynthesis J Chromatogr B Biomed Appl 655 179 87 8081463 10.1016/0378-4347(94)00107-3

[b85-aci-3-45] Yoshida T Honda A Shoda J 1997 Short-term effects of 3-hydroxy-3-methylglutaryl-CoA reductase inhibitor on cholesterol and bile acid synthesis in humans Lipids 32 873 8 9270980 10.1007/s11745-997-0112-2

[b86-aci-3-45] Yoshida T Honda A Matsuzaki Y 1999 Plasma levels of mevalonate and 7α-hydroxy-4-cholesten-3-one in chronic liver disease J Gastroenterol Hepatol 14 150 5 10029296 10.1046/j.1440-1746.1999.01819.x

